# GSK‐3β and mTOR Phosphorylation Mediate the Reversible Regulation of Hypomagnetic Field on Adult Neural Stem Cell Proliferation

**DOI:** 10.1111/ejn.70202

**Published:** 2025-07-17

**Authors:** Jie Ren, Fulai Li, Jianxun Shen, Lanxiang Tian, Yongxin Pan

**Affiliations:** ^1^ Biogeomagnetism Group, Key Laboratory of Planetary Science and Frontier Technology, Institute of Geology and Geophysics Chinese Academy of Sciences Beijing China; ^2^ College of Earth and Planetary Sciences University of Chinese Academy of Sciences Beijing China; ^3^ University of Chinese Academy of Sciences Beijing China; ^4^ Institute of Drug Discovery Technology Ningbo University Ningbo China

**Keywords:** adult neural stem cells, GSK‐3β, hypomagnetic field, mTOR, protein phosphorylation

## Abstract

Exposure to hypomagnetic field (HMF) of < 5 μT has been demonstrated to impair cognitive behaviors in mammals by disrupting neurogenesis. This process could potentially be modulated by the protein phosphorylation of adult neural stem cells (aNSCs) that are highly sensitive to environmental changes. However, the effects of HMF on aNSCs protein phosphorylation remain unclear. Here, we found that HMF reversibly regulates the effects on aNSC proliferation by modulating protein phosphorylation in aNSCs. Specifically, HMF inhibits aNSCs proliferation by reducing glycogen synthase kinase 3β (GSK‐3β) phosphorylation, and when aNSCs are returned from HMF to the geomagnetic field (rGMF), rGMF activates mammalian target of rapamycin (mTOR) phosphorylation to restore their proliferation. These findings not only advance our understanding of the molecular basis of HMF‐induced biological effects but also illuminate potential therapeutic targets for maintaining neural homeostasis in extreme environments.

## Graphical abstract

Working model illustrates the effects of GMF, HMF, and rGMF on aNSC proliferation. HMF suppresses the proliferation of aNSCs by reducing the phosphorylation of GSK 3β, thereby increasing its activity. Conversely, rGMF compensates for this suppression by activating mTOR via increased mTOR phosphorylation.

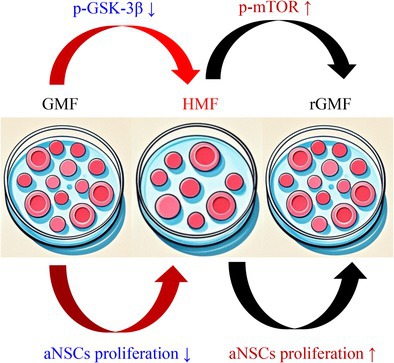

**Abbreviations**
aNPC/NSCs: adult neural progenitor cells/stem cells
CNS: central nervous system
DIA: data‐independent acquisition
DTT: dithiothreitol
GMF: geomagnetic field
GSK‐3β: glycogen synthase kinase 3β
HMF: hypomagnetic field
mTOR: mammalian target of rapamycin
PFA: paraformaldehyde
rGMF: returned to geomagnetic field
RT: room temperature

## 1 Introduction

The geomagnetic field (GMF) is fundamental to the Earth's biosphere and the evolution of life (Pan and Li 2023). However, with the development of human deep space exploration, astronauts will inevitably be exposed to the hypomagnetic field (HMF) environment with a magnetic field of < 5 μT (Afshinnekoo et al. 2020). It has previously been pointed out that HMF is one of the major environmental factors that interfere with the normal activity of the central nervous system (CNS) during spaceflight (Kokhan et al. 2016). Moreover, HMF can affect cognitive processes in humans as well as adult hippocampal neurogenesis and cognitive behaviors in mice (Sarimov et al. 2008; Zhang et al. 2021). Among different cell types in the CNS, adult neural progenitor cells/stem cells (aNPC/NSCs) are especially crucial for maintaining brain homeostasis and plasticity, which will exert influence on learning, memory, and emotional behaviors (Gonçalves et al. 2016). Recently, an in vivo study demonstrated that long‐term exposure (8 weeks) to HMF inhibited the proliferation and differentiation of adult hippocampal aNSCs in mice, but the inhibitory effects could be reversed when returned to the GMF condition (Zhang et al. 2021). However, the underlying mechanism remains obscure.

Mammal cells receive signals from the environment and respond to them. However, most key events involved in cellular responses are mediated by changes in posttranslational protein modifications rather than transcriptional changes (Olsen et al. 2006). Therefore, the effects of HMF exposure on aNSCs proliferation can be reversible, indicating the potential for a posttranslational modification. In cellular processes, protein phosphorylation can reversibly and dynamically regulate metabolism and growth as a prevalent posttranslational modification (Olsen et al. 2006; Pawson and Scott 2005). Previous studies have mainly focused on the effects of medium‐ to high‐intensity magnetic fields (static magnetic field, SMF) and electromagnetic fields (EMF). For example, SMF can activate or reduce the phosphorylation levels of multiple kinases and proteins (Halicka et al. 2009; Na et al. 2022). EMF can also regulate various cellular processes by modulating protein phosphorylation (Hu et al. 2001; Sun et al. 2008). However, the effect of HMF on protein phosphorylation in neural stem cells remains unclear. A recent study has found that protein phosphorylation in aNSCs can directly modulate neurogenesis (Zhao et al. 2024). Therefore, we propose that phosphorylation is a responsive mechanism for aNSCs when exposed to HMF. Understanding this process could provide new insights into how HMF inhibits neurogenesis in mice.

Therefore, this study aimed to determine if and how HMF exposure affects protein phosphorylation in aNSCs. First, data‐independent acquisition (DIA) phosphoproteomics showed that HMF could affect protein phosphorylation in aNSCs and that this effect was reversible. Further experimental validation revealed that HMF exposure activates glycogen synthase kinase 3β (GSK‐3β) activity by reducing phosphorylation levels (p‐GSK‐3β), thereby inhibiting aNSC proliferation. Notably, the GSK‐3β activity inhibitor Wnt3a was able to rescue the HMF‐mediated suppression of aNSC proliferation. Interestingly, when HMF‐exposed aNSCs were returned to GMF (rGMF), the inhibitory effects of HMF on aNSC proliferation were reversed by increased mammalian target of rapamycin (mTOR) phosphorylation (p‐mTOR). This rGMF‐mediated recovery was blocked by the mTOR inhibitor rapamycin. Our results demonstrate that GSK‐3β and mTOR phosphorylation play critical roles in mediating the reversible proliferative effects of HMF on aNSCs.

## 2 Materials and Methods

### 2.1 Isolation and Culturing of aNSCs

The aNSCs used in this study were isolated from the dentate gyrus of 8‐week‐old male mice using published methods (Guo et al. 2012). The experiment was carried out at the Institute of Genetics and Developmental Biology, Chinese Academy of Sciences. The cells were maintained in the IPM (neurobasal medium containing 2% B27; GIBCO, 17504‐044, United States), 2‐mM L‐glutamine (GIBCO, 25030081, United States), 20 ng/mL basic fibroblast growth factor (FGF‐2, PeproTech, K1606, United States), 20 ng/mL epidermal growth factor (EGF, PeproTech, A2306, United States), and 1% antibiotic‐antimycotic (GIBCO, 15240062, United States); plated in one well of a 24‐well dish; and cultured in a 5% CO2 incubator at 37°C. Half of the medium was replaced every 2 days.

### 2.2 Setups of Magnetic Field Conditions and Exposure Experiments

A 20‐layer nanocrystalline shielding bucket (central acrylic plate for dish placement) maintained HMF conditions (HMF: 117.26 ± 43.92 nT). The shielding bucket was placed in an incubator (Heal Force, HF240, China). The culture dishes for the control group (GMF) were placed next to the shielding bucket (GMF: 55,310.87 ± 280.94 nT) (Figure S1A–C). The magnetic field was monitored with an axial fluxgate magnetometer (Bartington, Spectramag‐6, United Kingdom).

### 2.3 aNSCs Proliferation Assay by EdU Labeling

EdU (10 μM) was added to the culture medium and incubated for 2 h at 37°C under 5% CO_2_. After labeling, the cells were washed twice with PBS and fixed with 4% paraformaldehyde (PFA) for 15 min at room temperature (RT). Permeabilization was performed using 0.5% Triton X‐100 in PBS for 20 min. The Click Reaction mixture was prepared according to the manufacturer's instructions (Beyotime, C0081L, China). Cells were incubated with the mixture for 30 min at RT in the dark. After three washes with PBS, nuclei were counterstained with Hoechst 33342 for 10 min.

### 2.4 Protein Extraction and Enzymatic Hydrolysis

Proteins were extracted using a lysis buffer containing 1× protease inhibitor cocktail and 10‐mM dithiothreitol (DTT), followed by centrifugation (25,000 g, 15 min, 4°C). After reduction (56°C, 1 h) and alkylation (55‐mM iodoacetamide, dark, 45 min), the supernatants were collected. One‐hundred micrograms of proteins was digested with trypsin (37°C, 4 h) after NH_4_HCO_3_ dilution. The extracted proteins were then desalted (Strata X column) and dried.

### 2.5 Enrichment of Phosphorylated Peptides and High pH Reversed‐Phase Separation

Phosphopeptides were enriched using zirconium oxide StageTips by sequential 400‐g centrifugation with Beijing Genomics Institute (BGI) buffers (wash with buffer 1, 50 μL, three times; washes with buffer 2, 80 μL, once; washes with buffer 3, 80 μL, once) and eluted twice with ammonia buffer. Pooled peptides were separated on a Gemini C18 column (Shimadzu LC‐20AB) using a 54‐min gradient (5%–95% mobile phase B) at 1 mL/min, monitored at 214 nm. Fractions collected every minute were pooled into 10 groups and lyophilized.

### 2.6 DIA‐Based Quantitative Detection

The lyophilized peptide samples were reconstituted in mobile phase A (2% ACN and 0.1% FA) and then centrifuged at 20,000 g for 10 min. The resulting supernatant was injected for analysis. Peptide separation was performed using a Thermo Ultimate 3000 UHPLC system. The separated peptides were ionized via a CSI nanoelectrospray source and analyzed in DIA mode on a timsTOF Pro tandem mass spectrometer. The key parameters were ion source voltage, 1.6 kV; ion mobility range, 0.75–1.40 V·s/cm^2^; and MS1 scan range, 100–1700 m/z. For DIA, the *m*/*z* range of 428–1128 was divided into four steps, each of which was further divided into seven windows (for a total of 28 windows), for sequential window fragmentation and data acquisition using CID fragmentation at a collision energy of 10 eV. Each window had a mass width of 25 Da, resulting in a DIA cycle time of 0.85 s.

### 2.7 DIA Data Analysis

DIA data are analyzed using DIA‐NN (false discovery rate < 1%) (Steger et al. 2021). Phosphorylation sites with localization probability < 0.75 and retained phosphorylation sites with high confidence are considered. A two‐sided Student's *t* test model was used to test the significance of phosphorylated peptides. Significant differences were defined as fold‐change > 2 and *p* < 0.05.

### 2.8 RNA Isolation and Quantitative Real‐Time PCR

RNA isolation was performed using Trizol (Invitrogen, 15596018, United States) based on the manufacturer's protocol. The first‐strand cDNA was generated by reverse transcription with an oligo (dT) primer (Promega, A5001, United States). Standard RT‐PCR was performed using GoTaq DNA polymerase (Promega, M3001, United States). To quantify the mRNA levels using real‐time PCR, aliquots of first‐stranded cDNA were amplified with gene‐specific primers and SYBR Green PCR Master Mix (Cwbiotech, CW0682A, China) using a Bio‐Rad Real‐Time PCR System. The sequences of primers used for PCR reactions were listed as follows: GSK‐3β, sense: 5′‐GACTTTGGAAGTGCAAAGC‐3′, antisense: 5′‐AGGAAATATTGGTTGTCCTAGC‐3′; mTOR, sense: 5′‐CTGATCCTCAACGAGCTAGTTC‐3′, antisense: 5′‐GGTCTTTGCAGTACTTGTCATG‐3′; Gapdh, sense: 5′‐AATGGGAAGCTTGTCATCAACG‐3′, antisense: 5′‐GAAGACACCAGTAGACTCCACGACATA‐3′.

### 2.9 Western Blot

The aNSCs were lysed in RIPA buffer (20‐mM Tris–HCl pH 7.5, 100‐mM NaCl, 0.1% SDS, 0.5% sodium deoxycholate, and 1‐mM PMSF) containing Complete Protease Inhibitor Cocktail (Roche, 04693132001, Switzerland) and phosphatase Inhibitor Cocktail (Roche, 04906845001, Switzerland). Cell lysates were centrifuged at 12,000 rpm for 10 min. Fifty micrograms of supernatants were resolved on SDS‐PAGE and blotted with the indicated antibodies. Primary antibodies used were rabbit anti‐Phospho‐GSK‐3β (1:1000, Cell Signaling, 9323, United States), rabbit antiphospho‐mTOR (1:1000, Signaling, 2974, United States), mouse anti‐GSK‐3β (1:500, Abcam, ab93926, United States), mouse anti‐mTOR (1:1000, Cell Signaling, 4517S, United States), and rabbit antibeta Tubulin (1:1000, Abcam, ab179513, United States). The amount of beta Tubulin was used as the loading control. Secondary antibodies used were Goat antirabbit IgG H&L (HRP) (1:10000, Abcam, ab205718, United States) and goat antimouse IgG H&L (HRP) (1:5000, Abcam, ab205719, United States).

### 2.10 Statistical Analysis

Data were analyzed using GraphPad Prism 8.3 software, and significant differences between groups were determined by unpaired Student's *t* test and one‐way ANOVA. Results were considered significant when *p* values < 0.05. All data were shown as mean with standard error of mean (mean ± SEM).

## 3 Results

### 3.1 HMF Modulation of Protein Phosphorylation in aNSCs

**FIGURE 1:**
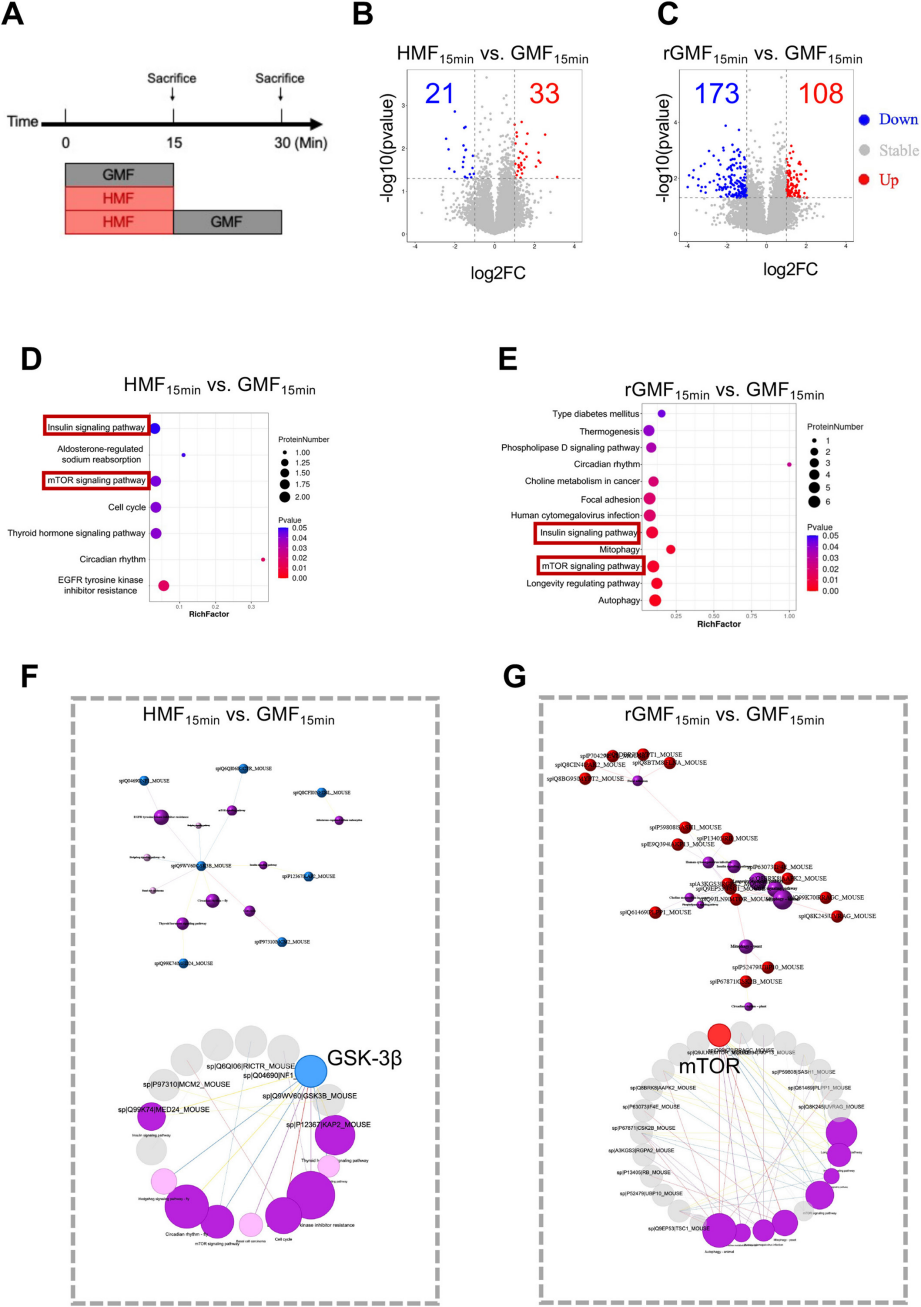
Analysis of the number of differentially phosphorylated peptides. (A) Experimental timeline for DIA phosphoproteomics of aNSCs during GMF, HMF, or rGMF exposure. (B, C) Volcano plots. The red dot indicates significantly upregulated, the green dot indicates significantly downregulated, and the gray dot indicates nonsignificant change. (D, E) Significantly enriched pathway. This figure shows the metabolic pathway in which the differentially expressed proteins are significantly enriched. The *X* axis is the enrichment factor (RichFactor), which represents the number of differential proteins annotated to the pathway divided by all the proteins identified in the pathway. Larger values indicate the greater proportions of differential proteins annotated to the pathway. The size of the circle represents the number of differential proteins annotated to the pathway. (F, G) Pathway relationship networks. Red dots represent upregulated proteins, and the blue dots represent the downregulated proteins. The purple circles represent the top 10 enriched pathways, darker purple indicates significant enrichment, lighter purple indicates insignificant enrichment, and a larger area indicates a higher level of enrichment.

To investigate the effects of HMF on protein phosphorylation, we first performed DIA phosphoproteomic analysis on aNSCs exposed to GMF, HMF, and rGMF for 15 min (Figure 1A). The results are presented in Figure 1B; compared to the GMF_15min_ group, the 15 min exposure to HMF (HMF_15min_) induced 54 differentially phosphorylated peptides in aNSCs, with 33 upregulated and 21 downregulated phosphorylated peptides. Compared to GMF_15min_, aNSCs in rGMF_15min_ exhibited 281 differentially phosphorylated peptides, including 108 upregulated phosphorylated peptides and 173 downregulated phosphorylated peptides (Figure 1C).

We then performed pathway enrichment analysis on proteins corresponding to the differentially phosphorylated peptides. Interestingly, the “insulin signaling pathway” and the “mTOR signaling pathway” were simultaneously found in both the HMF‐downregulated phosphopeptide‐enriched pathways and the rGMF‐upregulated phosphopeptide‐enriched pathways (Figure 1D,E). Further analysis of the pathway relationship networks revealed that decreased GSK‐3β phosphorylation served as a major contributor to the downregulated signaling pathways after 15 min of HMF exposure (Figure 1F), whereas the upregulated phospho‐signaling pathways upon GMF restoration were all associated with increased mTOR phosphorylation (Figure 1G). These data demonstrate that HMF‐induced changes in aNSC phosphoproteomic profiles are partially restored upon GMF restoration, with GSK‐3β and mTOR potentially playing key roles during the HMF exposure and GMF restoration phases, respectively.

### 3.2 HMF Reduces GSK‐3β Phosphorylation and Inhibits aNSC Proliferation

**FIGURE 2:**
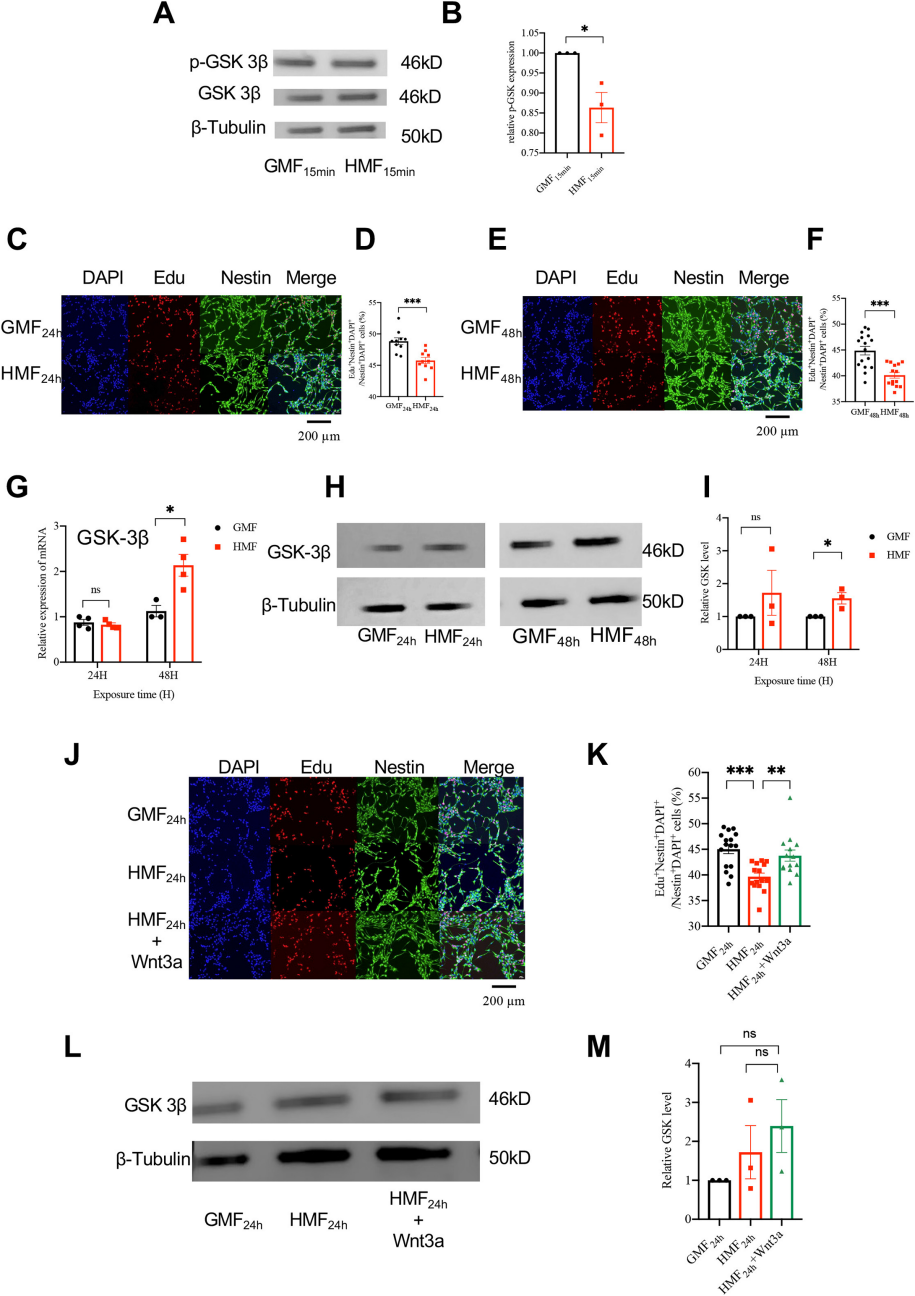
HMF reduces GSK‐3β phosphorylation and inhibits aNSC proliferation. (A) Western blot analysis of GSK‐3β phosphorylation levels in aNSCs after 15 min exposure to GMF and HMF. (B) Quantification of GSK‐3β phosphorylation levels in aNSCs after 15‐min exposure to GMF and HMF. (C) Representative images of aNSC proliferation after 24‐h exposure to GMF and HMF. Scale bar = 200 μm. (D) Percentage of Edu^+^ aNSCs in total aNSCs after 24 h of exposure to HMF and GMF. (E) Representative images of aNSC proliferation after 48 h of exposure to GMF and HMF. Scale bar = 200 μm. (F) Percentage of Edu^+^ aNSCs out of total aNSCs after 48‐h exposure to HMF and GMF. (G) Relative GSK‐3β mRNA expression levels in aNSCs after 24 and 48 h of HMF and GMF exposure. (H) Western blot analysis of relative GSK‐3β expression levels in aNSCs after 24 and 48 h of HMF and GMF exposure. (I) Quantification of relative GSK‐3β expression levels in aNSCs after 24 and 48 h of HMF and GMF exposure. (J) Representative images of aNSC proliferation after 24‐h exposure to GMF, HMF and HMF + Wnt3a. Scale bar = 200 μm. (K) Percentage of Edu^+^ aNSCs in total aNSCs after 24‐h exposure to GMF, HMF, and HMF + Wnt3a. (L) Western blot analysis of relative GSK‐3β expression levels in aNSCs after 24 h of exposure to HMF, GMF, and HMF + Wnt3a. (M) Quantification of relative GSK‐3β expression levels in aNSCs after 24 and 48 h of exposure to HMF, GMF, and HMF + Wnt3a. *n* = 3. Data are presented as the mean ± SEM; *p < 0.05, **p < 0.05, and ***p < 0.001.

To validate the HMF‐induced effects on GSK‐3β identified in the DIA proteomic analysis, we measured GSK‐3β phosphorylation levels in aNSCs after 15‐min exposure to either GMF or HMF. Compared to GMF controls, HMF exposure significantly reduced GSK‐3β phosphorylation (Figure 2A,B). Reduced inhibitory phosphorylation (Ser9) activates GSK‐3β, which phosphorylates β‐catenin for proteasomal degradation (Grimes and Jope 2001; Plyte et al. 1992). This mechanism represses the Wnt pathway and ultimately inhibits cell cycle progression (Peifer and Polakis 2000). Phosphorylation acts as a rapid molecular switch for protein function on the scale of seconds to minutes (Olsen et al. 2006). However, cellular proliferation results from a gradual amplification of signaling cascades triggered by phosphorylation. This process involves multiple steps, including DNA synthesis, chromosome replication, and mitosis, all of which require significant time (Ligasová et al. 2023). Consequently, we evaluated cell proliferation at 24 and 48 h after HMF exposure, ensuring that sufficient time had been allowed for the completion of the cell cycle. Our results showed that 24 and 48 h of HMF treatment significantly inhibited the proliferation of primary aNSCs cultured in vitro (Figure 2C–F). We also analyzed GSK‐3β expression at both mRNA and protein levels. No significant differences were observed between HMF‐ and GMF‐exposed groups after 24 h. However, extending HMF exposure to 48 h resulted in a significant upregulation of GSK‐3β mRNA and protein expression compared to GMF controls (Figure 2G–I). These results suggest that HMF‐induced dysregulation of GSK‐3β (phosphorylation, mRNA, and protein) impaired aNSC proliferation.

To further investigate whether HMF inhibits the proliferation of aNSCs by activating GSK‐3β, in the classical Wnt/β‐catenin pathway, activation of the Wnt signaling pathway can inhibit GSK‐3β activity (Grimes and Jope 2001). We administered Wnt3a during HMF exposure and assessed cell proliferation along with GSK‐3β protein expression. The results showed that the proliferation of aNSCs with added Wnt 3a upon exposure to HMF was significantly higher than that of the HMF group and was consistent with the proliferation of aNSCs in GMF (Figure 2J,K). Notably, total GSK‐3β protein levels showed no significant differences across conditions (Figure 2L,M). These findings demonstrate that Wnt3a rescues the HMF‐induced suppression of cell proliferation.

Taken together, the above results supported that HMF inhibits the proliferation of aNSCs by reducing the phosphorylation of GSK‐3β.

### 3.3 rGMF Increases mTOR Phosphorylation and Restores aNSC Proliferation

**FIGURE 3:**
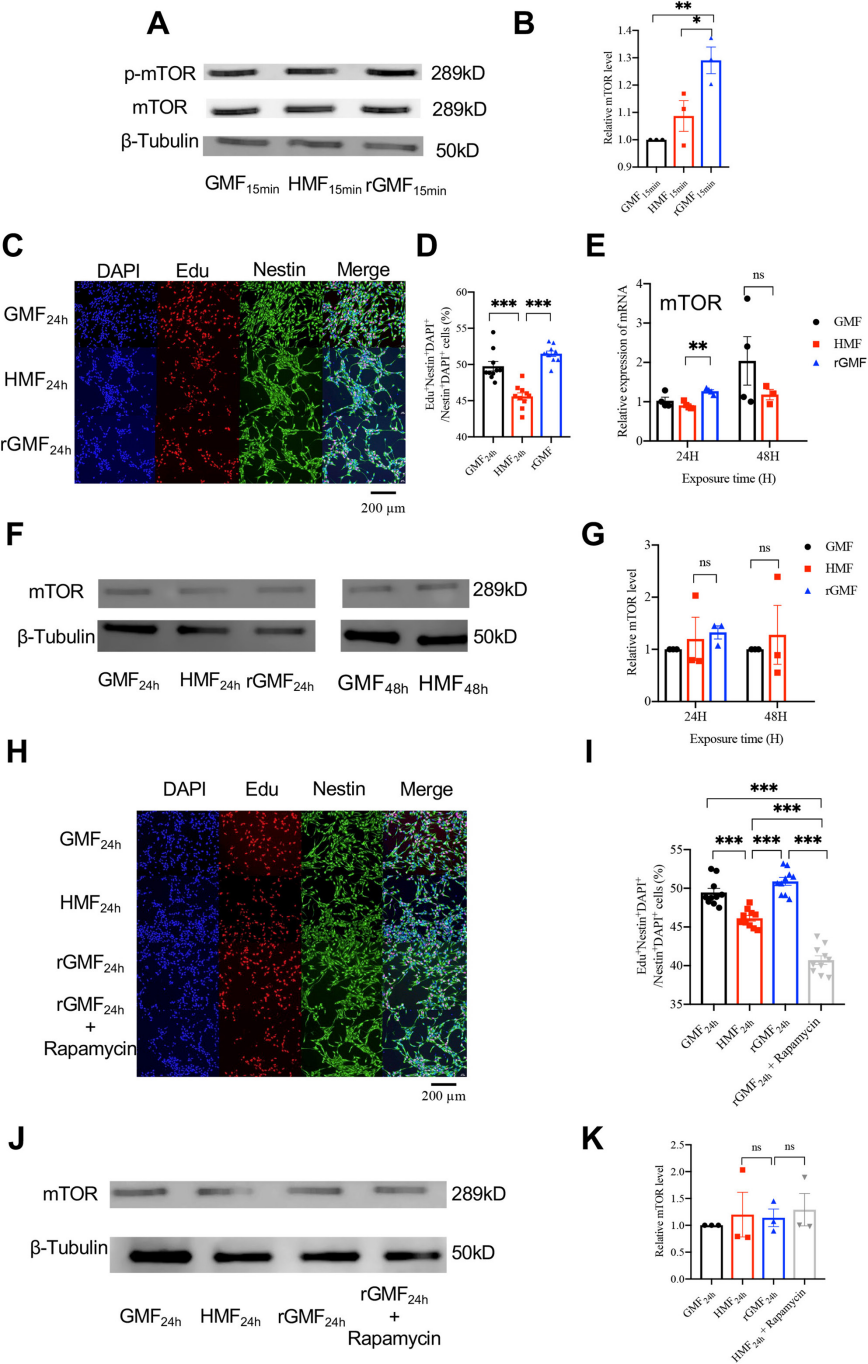
rGMF increases mTOR phosphorylation and restores aNSC proliferation. (A) Western blot analysis of mTOR phosphorylation levels in aNSCs after 15‐min exposure to GMF, HMF, and rGMF. (B) Quantification of mTOR phosphorylation levels in aNSCs after 15‐min exposure to GMF, HMF, and rGMF. (C) Representative images of aNSC proliferation after 24‐h exposure to GMF, HMF, and rGMF. Scale bar = 200 μm. (D) Percentage of Edu^+^ aNSCs in total aNSCs after 24 h of exposure to GMF, HMF, and rGMF. (E) Relative mTOR mRNA expression levels in aNSCs after 24 and 48 h of exposure to GMF, HMF, and rGMF. (F) Western blot analysis of relative mTOR expression levels in aNSCs after 24 and 48 h of exposure to HMF and GMF. (G) Quantification of relative mTOR expression levels in aNSCs after 24 and 48 h of HMF and GMF exposure. (H) Representative images of aNSC proliferation after 24‐h exposure to GMF, HMF, rGMF, and rGMF + Rapamycin. Scale bar = 200 μm. (I) Percentage of Edu^+^ aNSCs in total aNSCs after 24 h of exposure to GMF, HMF, rGMF, and rGMF + Rapamycin. (J) Western blot analysis of mTOR phosphorylation levels in aNSCs after 15‐min exposure to GMF, HMF, rGMF, and rGMF + Rapamycin. (K) Quantification of relative mTOR expression levels in aNSCs after 24 and 48 h of exposure to GMF, HMF, rGMF, and rGMF + Rapamycin. *n* = 3. Data are presented as the mean ± SEM; *p < 0.05, **p < 0.01, and ***p < 0.001.

DIA proteomics results of rGMF showed that when aNSCs were returned to GMF after exposure to HMF, the phosphorylation level of mTOR was increased. To validate these results, aNSCs were exposed to GMF, HMF, and rGMF for 15 min. The results showed that the phosphorylation level of mTOR after 15 min of HMF exposure showed no significant difference compared to the GMF_15min_ group. However, in the rGMF group, mTOR protein phosphorylation levels were significantly higher than those in both the GMF_15min_ and HMF_15min_ groups (Figure 3A,B). As mTOR regulates various fundamental physiological processes, including cell proliferation (Saxton and Sabatini 2017), we also investigated the proliferative capacity of aNSCs after 24‐h exposure to rGMF. The results showed that aNSC proliferation was significantly higher in the rGMF_24h_ group than in the HMF_24h_ group, but not different from the GMF_24h_ group (Figure 3C,D). Furthermore, detection of mTOR mRNA and protein expression levels revealed no differences in mRNA levels after 24 and 48 h of HMF exposure. However, when aNSCs were returned to GMF (rGMF_24h_), mTOR mRNA levels increased significantly compared to HMF_24h_, while remaining comparable to the GMF_24h_ group (Figure 3E). Protein expression of mTOR levels showed no significant differences between the HMF, HMF, and rGMF groups (Figure 3F,G). These results suggest that mTOR protein phosphorylation may be associated with the restorative effects on cell proliferation during rGMF exposure.

To further investigate whether rGMF restores cell proliferation by activating the mTOR pathway, we administered the mTOR‐specific inhibitor rapamycin when aNSCs were returned to GMF after HMF exposure (Chung et al. 1992). The results showed that rapamycin‐treated aNSCs exhibited significantly reduced proliferation compared to the rGMF group (Figure 3H,I). Notably, rapamycin treatment did not affect total mTOR protein expression (Figure 3J,K). These results suggest that rapamycin blocks rGMF‐mediated restoration of aNSC proliferation.

Taken together, the above results supported that rGMF increases mTOR phosphorylation and restores aNSC proliferation.

## 4 Discussion

Understanding the molecular mechanisms underlying mammalian responses to HMF is not only a critical issue in magnetobiology but also provides an important experimental basis for protecting the CNS during human space exploration. In this study, we found that HMF reversibly affects aNSC proliferation through the regulation of protein phosphorylation. This demonstrates the high sensitivity of aNSC protein phosphorylation to magnetic field variations. These findings are consistent with our in vivo animal experiments showing that HMF‐induced impairments in adult hippocampal neurogenesis can be reversed after 8 weeks of prolonged HMF exposure followed by 4 weeks of recovery under GMF conditions (Zhang et al. 2021). We therefore propose that the suppression of adult hippocampal neurogenesis by HMF may be mediated by early phosphorylation regulation of aNSC proteins. Changes in phosphorylation represent rapid signaling responses. Environmental stimuli can quickly regulate the phosphorylation status of proteins over short periods, constituting an early adaptive cellular response (Olsen et al. 2006). Conversely, cell proliferation is a downstream effect that accumulates over time (Ligasová et al. 2023). Therefore, changes in phosphorylation serve as the upstream point of signal transduction, whereas inhibition of proliferation is the downstream phenotypic outcome.

Our results demonstrate that the inhibitory effect of HMF on aNSC proliferation is mediated through suppression of GSK‐3β phosphorylation, which activates GSK‐3β kinase activity and thereby inhibits cell growth. GSK‐3β, a phylogenetically conserved serine/threonine kinase abundantly expressed in the CNS, plays critical regulatory roles in cellular differentiation, proliferation, survival, apoptosis, energy metabolism, morphogenesis, and neural development (Leroy and Brion 1999; Lin et al. 2020). Dysregulation of GSK‐3β has been implicated in several pathologies including cancer, neurodegenerative, and neuropsychiatric disorders (Gianferrara et al. 2022; Lauretti et al. 2020). Our experimental data showed that short‐term exposure to HMF (15 min) significantly reduced GSK‐3β phosphorylation levels. Prolonged exposure (48 h) further induced a marked upregulation of total GSK‐3β protein expression. Mechanistically, HMF‐induced dephosphorylation triggered GSK‐3β activation. Functionally, activated GSK‐3β acts as a negative regulator of the Wnt/β‐catenin signaling pathway, a key driver of proliferative processes (Dale 1998). This inhibitory effect was supported by rescue experiments showing that Wnt3a supplementation effectively counteracted HMF‐mediated suppression of cell proliferation. Collectively, our findings elucidate a novel molecular pathway by which HMF exerts its antiproliferative effects through precise modulation of GSK‐3β activity.

Interestingly, we observed that when sNSCs were returned to GMF conditions after HMF exposure, recovery from HMF‐induced suppression of cell proliferation was mediated by increased mTOR phosphorylation. The target of rapamycin (TOR), a highly conserved serine/threonine protein kinase belonging to the PI3K‐related kinase family, serves as a critical signaling hub in mammalian cells. mTOR regulates essential physiological processes, including cell growth, proliferation, metabolism, and survival (Saxton and Sabatini 2017). Dysregulation of mTOR signaling has been implicated in several major diseases, including diabetes, cancer, and neurological disorders (Lipton and Sahin 2014; Saxton and Sabatini 2017). Our study showed that a 15‐min exposure to HMF and return to GMF activated mTOR phosphorylation in aNSCs, effectively rescuing the HMF‐induced suppression of aNSC proliferation. Significantly, when the mTOR inhibitor rapamycin was administered during GMF restoration, the recovery of proliferative capacity in rGMF‐treated aNSCs was completely abolished. These results establish that the proliferative recovery observed during GMF restoration is mechanistically dependent on mTOR‐mediated signaling pathways.

Our study found that reducing the magnetic field strength (HMF) decreases the phosphorylation of GSK‐3β, thereby activating it. This subsequently leads to the inhibition of the Wnt signaling pathway, ultimately suppressing cell cycle progression. Conversely, increasing magnetic field strength (rGMF) elevates the phosphorylation level of mTOR, thereby restoring inhibited cell proliferation. In summary, HMF and rGMF regulate the biological effects of magnetic field strength on cell proliferation by rapidly modulating the phosphorylation of GSK‐3β and mTOR. Interestingly, the GSK‐3β and mTOR signaling pathways are closely interconnected in the regulation of cellular proliferation. These pathways play a key role in controlling cell proliferation and growth, interacting in a complex and intricate way that enables precise control of cell fate (McCubrey et al. 2016). Active GSK‐3β promotes the function of the TSC complex, thereby inhibiting mTOR activity. This represents negative regulation of mTOR by GSK‐3β. Inhibiting mTOR subsequently restricts protein synthesis and cell growth/proliferation (Hur and Zhou 2010). Conversely, mTOR can also regulate GSK‐3β activity to modulate proliferation. mTOR‐mediated regulation of cellular proliferation is orchestrated by its activation of the downstream Akt signaling pathway (Saxton and Sabatini 2017). Interestingly, Akt activation is required for GSK‐3β inactivation. Previous studies show that Akt serves as a key effector in insulin/PI3K signaling (Sarbassov et al. 2005), with the PI3K/Akt pathway predominantly repressing GSK‐3β in various contexts (Grimes and Jope 2001; Park et al. 1999). Activated Akt promotes cell survival, proliferation, and growth by phosphorylating and inhibiting critical substrates, including GSK‐3β. We therefore hypothesize that GSK‐3β activity may be similarly regulated during rGMF‐enhanced aNSC proliferation mediated by increased mTOR phosphorylation, and we hope to confirm this in our subsequent studies.

## 5 Conclusion

In summary, our study demonstrates the reversibility of HMF effects on aNSCs. Specifically, HMF suppresses the proliferation of aNSCs by reducing the phosphorylation of GSK 3β, thereby increasing its activity. Conversely, restoration of rGMF compensates for this suppression by activating mTOR via increased mTOR phosphorylation. These findings not only advance our understanding of the molecular basis of HMF‐induced biological effects but also illuminate potential therapeutic targets for maintaining neural homeostasis in extreme environments.

## Author Contributions

**Jie Ren:** conceptualization, formal analysis, methodology, validation, writing – original draft, writing – review and editing. **Fulai Li:** formal analysis, methodology, validation. **Jianxun Shen:** conceptualization, validation, writing – review and editing. **Lanxiang Tian:** conceptualization, funding acquisition, supervision, writing – review and editing. **Yongxin Pan:** funding acquisition, supervision, writing – review and editing.

## Ethics Statement

All procedures and husbandry were performed according to protocols approved by the Institutional Animal Care and Use Committee at the Institute of Genetics and Developmental Biology, Chinese Academy of Sciences.

## Conflicts of Interest

The authors declare no conflicts of interest.

## Peer Review

The peer review history for this article is available at https://www.webofscience.com/api/gateway/wos/peer‐review/10.1111/ejn.70202.

## Supporting information


**Figure S1** **Magnetic field setups.** (**A**) A nanocrystalline material was designed to maintain a HMF condition. The magnetic shielding bucket is a cylinder with a length of 40 cm and a diameter of 14 cm. It consists of 20 layers of nanocrystalline material, with acrylic plates positioned in the center to support the culture dishes. The culture dishes for the control group (GMF) are positioned adjacent to the shielding bucket. (**B**) The intensity of the GMF was 55,310.87 ± 280.94 nT. (**C**) The intensity of the HMF was 117.26 ± 43.92 nT.

**Data S1** Supporting Information.

## Data Availability

The datasets are available from the corresponding author on request.
