# Supplementary material for: GSK‐3β and mTOR Phosphorylation Mediate the Reversible Regulation of Hypomagnetic Field on Adult Neural Stem Cell Proliferation
Source: Eur J Neurosci. 2025 Jul 17;62(2):e70202. doi: 10.1111/ejn.70202 (PMC12271677; doi:10.1111/ejn.70202)
Supplement: Supplementary file 1 — Figure S1 Magnetic field setups. (A) A nanocrystalline material was designed to maintain a HMF condition. The magnetic shielding bucket is a cylinder with a length of 40 cm and a diameter of 14 cm. It consists of 20 layers of nanocrystalline material, with acrylic plates positioned in the center to support the culture dishes. The culture dishes for the control group (GMF) are positioned adjacent to the shielding bucket. (B) The intensity of the GMF was 55,310.87 ± 280.94 nT. (C) The intensity of the HMF was 117.26 ± 43.92 nT. [file EJN-62-0-s002.docx]

**Supplementary Figure 1**

**Supplementary figure legend**

**Supple****mentary Figure 1: Magnetic field setups.**

(**A**) A nanocrystalline material was designed to maintain a HMF condition. The magnetic shielding bucket is a cylinder with a length of 40 cm and a diameter of 14 cm. It consists of 20 layers of nanocrystalline material, with acrylic plates positioned in the center to support the culture dishes. The culture dishes for the control group (GMF) are positioned adjacent to the shielding bucket. (**B**) The intensity of the GMF was 55,310.87 ± 280.94 nT. (**C**) The intensity of the HMF was 117.26 ± 43.92 nT.
