# Supplementary material for: GSK‐3β and mTOR Phosphorylation Mediate the Reversible Regulation of Hypomagnetic Field on Adult Neural Stem Cell Proliferation
Source: Eur J Neurosci. 2025 Jul 17;62(2):e70202. doi: 10.1111/ejn.70202 (PMC12271677; doi:10.1111/ejn.70202)
Supplement: Supplementary file 2 — Data S1 Supporting Information. [file EJN-62-0-s001.pdf]

1. Full unedited blot for Figure 2A

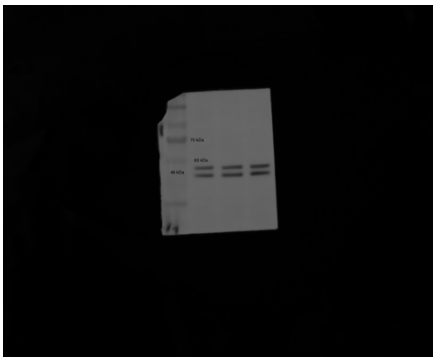

Fig. 2A GSK-3β+tubulin

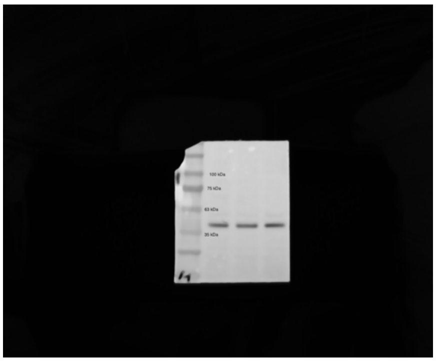

Fig. 2A p-GSK-3β

2. Full unedited blot for Figure 2H

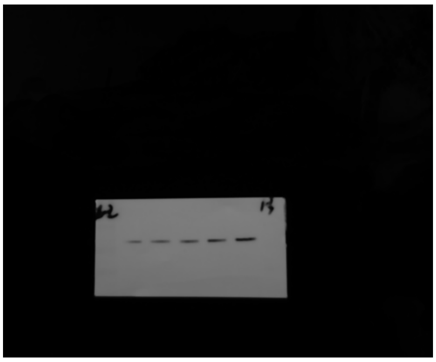

Fig. 2H GSK-3β

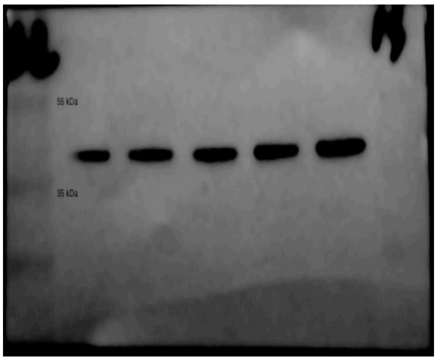

Fig. 2H GSK-3β marker

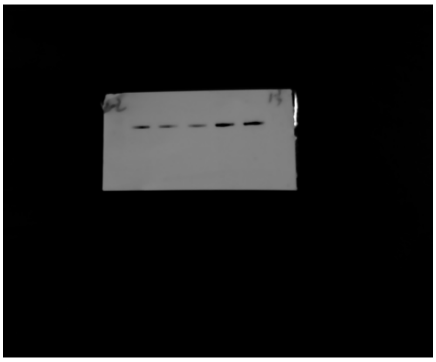

Fig. 2H tubulin

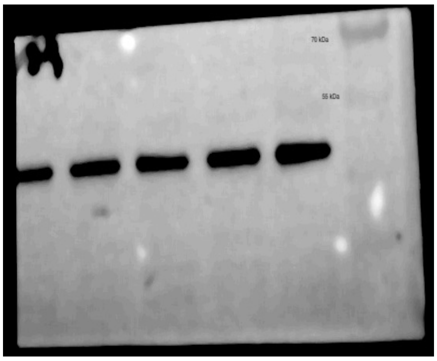

Fig. 2H tubulin marker

3. Full unedited blot for Figure 2L

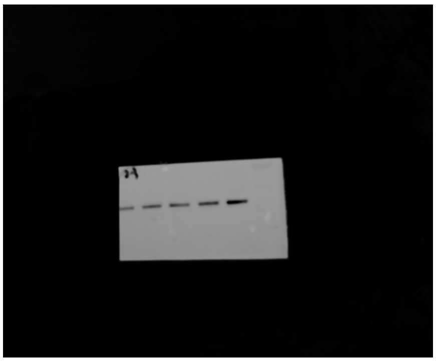

Fig. 2L GSK-3β

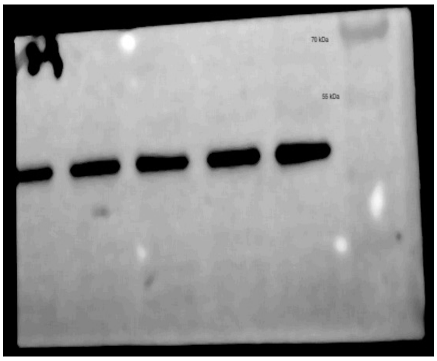

Fig. 2L GSK-3β marker

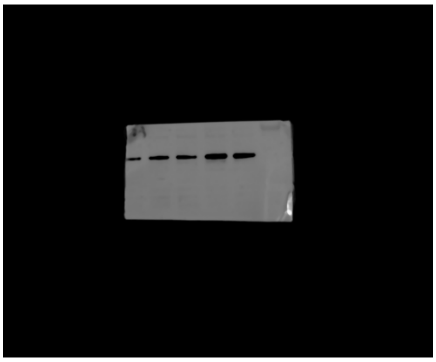

Fig. 2L tubulin

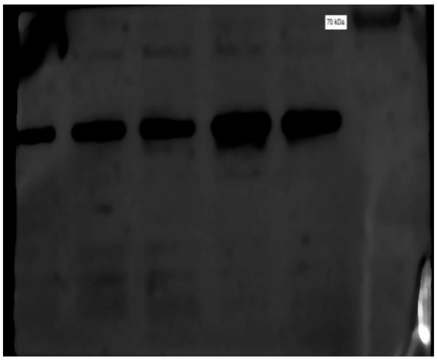

Fig. 2L tubulin marker

4. Full unedited blot for Figure 3A

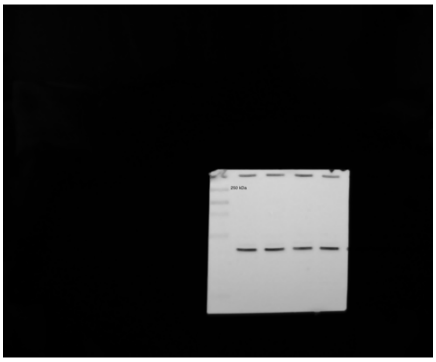

Fig. 3A p-mTOR

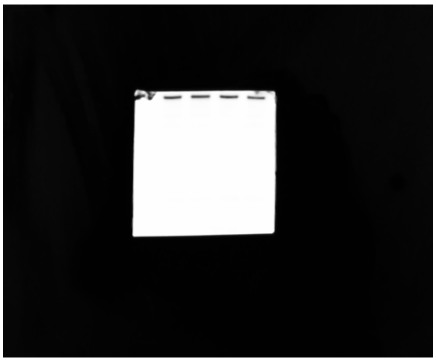

Fig. 3A mTOR

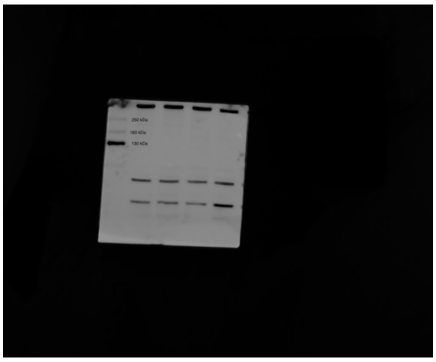

Fig. 3A tubulin

5. Full unedited blot for Figure 3F

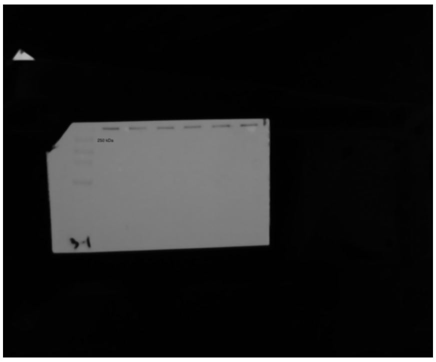

Fig. 3F mTOR

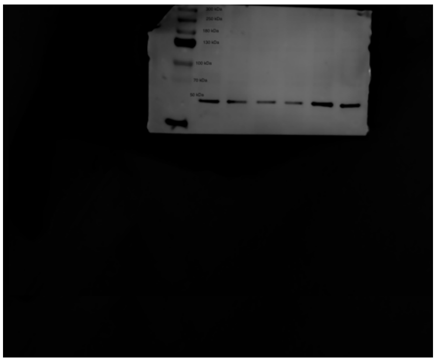

Fig. 3F tubulin

6. Full unedited blot for Figure 3J

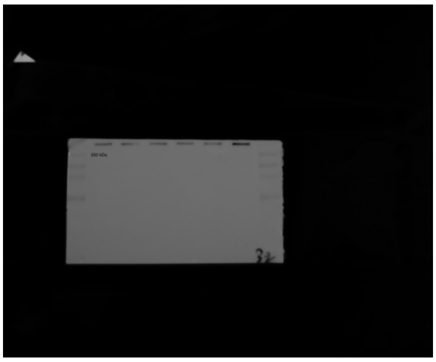

Fig. 3J mTOR

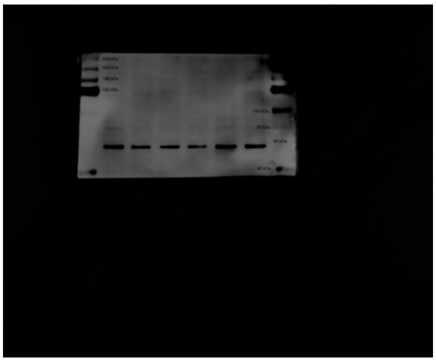

Fig. 3J tubulin
